# Ondansetron: a potential intervention for improving clinical outcomes in stroke patients

**DOI:** 10.3389/fphar.2025.1604117

**Published:** 2025-09-09

**Authors:** Jian Xu, Mengfei Zhang, Leyu Tao, Shuying Zhang, Zhihua Tang

**Affiliations:** Department of Pharmacy, Shaoxing People’s Hospital, Shaoxing, China

**Keywords:** ondansetron, stroke, mortality, propensity score matching, clinical outcomes

## Abstract

**Background:**

Stroke is a major global public - health problem. It is a cerebrovascular disease with sudden onset, high prevalence, and high rates of death and disability. Ondansetron (a 5 - HT3 receptor antagonist) an antiemetic, has recently been indicated in studies to have the ability to influence neurotransmitter imbalance, towardly have more effective against vomiting in stroke patients. However, its impact on stroke patients’ clinical outcomes remains unclear. This study uses real - world data to evaluate ondansetron’s effect on stroke patients’ clinical outcomes. Findings could lead to new treatments and better outcomes.

**Methods:**

This study was a retrospective cohort analysis involving adult patients who experienced a stroke, categorized into two groups: the ondansetron group and the non-ondansetron group. To ensure the baseline characteristics were balanced, propensity score matching (PSM) was utilized. The mortality rate was assessed using multivariable Cox regression models along with Kaplan-Meier survival curves. Additionally, subgroup analyses were performed to examine the consistency of the findings.

**Results:**

A total of 5,297 stroke patients were included in this study, among which 3,926 stroke patients received ondansetron treatment on the first day of admission to the intensive care unit (ICU), and 1,371 did not receive this drug treatment. After PSM, 2,628 patients were paired. The analysis results showed that the use of ondansetron on the first day of admission to the ICU significantly reduced the 30-day mortality rate (hazard ratio [HR] 0.73, 95% confidence interval [CI]: 0.59–0.92, P < 0.05). Meanwhile, the 60-day mortality rate also decreased significantly (HR 0.73, 95% CI: 0.60–0.90, P < 0.05).

**Conclusion:**

Treatment using ondansetron is linked to an enhancement in the overall prognosis for stroke patients. Those who are administered ondansetron on the initial day of their ICU admission experience a notably lower mortality rate. The results of this research provide a compelling and valuable addition to the conventional stroke treatment protocol, holding considerable clinical importance and scientific research relevance.

## Introduction

Stroke, a cerebrovascular disorder characterized by its sudden onset, represents a major public health concern worldwide. It ranks among the leading causes of mortality and long-term disability. The high incidence, mortality, and disability rates associated with stroke impose substantial burdens on patients and their families while also presenting significant challenges to healthcare systems. Current therapeutic strategies primarily aim at rapid restoration of cerebral blood flow, such as thrombolytic therapy and thrombectomy in cases of ischemic stroke, as well as the management of intracranial pressure and bleeding in hemorrhagic stroke cases (Hankey, 2017). Nevertheless, despite the application of optimal available treatments, many patients continue to experience adverse outcomes, including severe neurological impairments, cognitive difficulties, and an increased likelihood of stroke recurrence (Montellano et al., 2021).

Following the onset of a stroke, particularly in cases of ischemic stroke, ischemia and hypoxia are induced in the affected brain tissue, resulting in a significant release of 5-hydroxytryptamine (5-HT) from nerve endings and platelets (Bateman et al., 2016). As a potent vasoactive agent, the overactivation of 5-HT contributes to the exacerbation of vascular spasm. The selective antagonist ondansetron binds specifically to the 5-HT3 receptor, thereby inhibiting the interaction between 5-HT and its receptor (Naylor and Rudd, 1992). Consequently, cerebrovascular spasm induced by excessive 5-HT release is alleviated, aiding in the preservation of cerebral blood flow and reducing secondary neuronal damage in the ischemic penumbra region due to hypoperfusion. This mechanism is critically important for rescuing neurons that are at risk of death and for improving neurological function in stroke patients (Román et al., 2021).

Several studies have suggested that ondansetron may improve the prognosis for stroke patients. However, these studies have limitations. Some focus on the mechanism of action, neglecting its practical implications in clinical settings (Sharma et al., 2015). Others have investigated ondansetron’s clinical applications, but methodological challenges such as small sample sizes and inadequate study designs impede precise evaluation of its efficacy in stroke patients.

Therefore, this study investigates the pharmacological mechanism of ondansetron in the treatment of stroke patients. Utilizing the MIMIC-IV database, we aim to explore the association between ondansetron use and clinical outcomes in real-world scenarios. This database, derived from actual clinical practice, provides high-quality data that enhances the practical relevance of our study. Furthermore, propensity score matching (PSM) will be implemented to reduce baseline differences between treatment groups, approximating a randomized trial design and thereby enhancing the robustness and reliability of our findings.

## Methods

### Data source

The information for this study was obtained from the MIMIC-IV dataset. MIMIC-IV is an extensive, de-identified database focused on intensive care units (ICUs) that is publicly available. The information is collected from the electronic medical record system utilized in the Intensive Care Units (ICUs) at Beth Israel Deaconess Medical Center (BIDMC). These records provide comprehensive medical details of patients in actual ICU environments, covering multiple elements such as basic patient demographics, vital signs, laboratory results, and treatment procedures [Name] has successfully completed the evaluation and received permission to utilize the data.

### Participant selection

**FIGURE 1:**
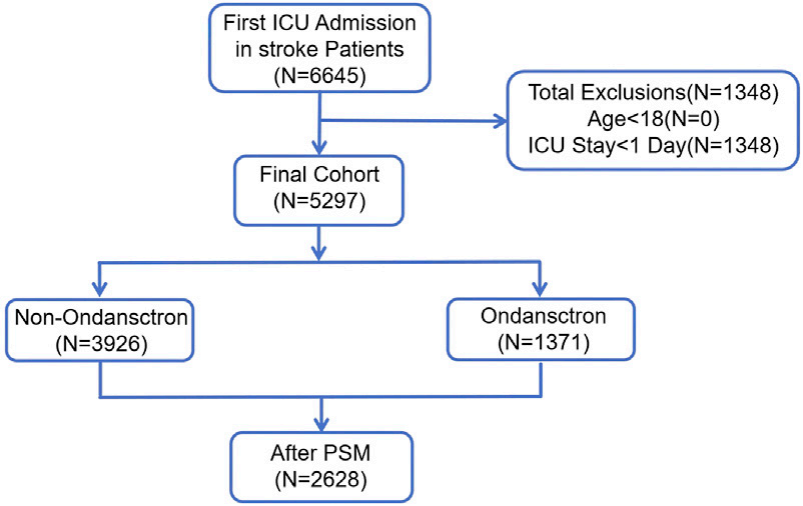
The flow chart of the study. MIMIC-IV, Medical Information Mart for Intensive Care IV; ICU, intensive care unit; PSM, propensity-score matching.

All information was gathered within 24 h following the patients’ admission to the hospital. Stroke patients were identified using ICD-9 and ICD-10 classifications. A total of 6,645 instances of initial ICU admissions among stroke patients were found. Those younger than 18 years of age (n = 0) and individuals who had an ICU stay shorter than 24 h (n = 1,348) were excluded. Ultimately, 5,297 patients qualified for the study. These individuals were categorized into two groups: 3,926 patients who were administered ondansetron during their ICU stay, and 1,371 patients who did not receive ondansetron. By applying Propensity Score Matching (PSM), a total of 2,628 patients were chosen for the final evaluation (1,314 patients in each group) (Figure 1).

### Variables

Demographic data included patients’ age, gender, and race. Collected vital signs and laboratory values comprised heart rate, systolic blood pressure (SBP), respiratory rate, peripheral capillary oxygen saturation (SpO_2_), Sequential Organ Failure Assessment (SOFA) score, Glasgow Coma Scale (GCS), Organ Dysfunction and Severity Index Score (OASIS), Logistic Organ Dysfunction Score (LODS), Charlson Comorbidity Index (CCI), red blood cell (RBC) count, platelet count, sodium level, potassium level, calcium level, blood urea nitrogen (BUN), creatinine level, and international normalized ratio (INR). Additionally, comorbidity information such as myocardial infarct, chronic pulmonary disease, peptic ulcer disease, acute kidney injury (AKI), sepsis, hypertension, dopamine use, and epinephrine use was gathered. The operational definitions and calculation methods for the key variables SOFA, GCS, and CCI are provided in Supplementary Table S1.

The intervention measures considered in this study included the use of vasoactive drugs (e.g., dopamine, epinephrine, norepinephrine, and neuroblockers), mechanical ventilation ratio (MV), and continuous renal replacement therapy (CRRT).

### Outcomes

The primary outcome was 30-day mortality, while the secondary outcome was 60-day mortality.

### Statistical analysis

Descriptive analysis was conducted for each participant. For continuous variables, data that followed a normal distribution were reported as mean ± standard deviation (SD), while data that exhibited a skewed distribution were reported as median and interquartile range (IQR). To compare differences between groups, the chi-square test was utilized for categorical variables. For continuous variables, the Student’s t-test was employed for normally distributed data, and the Mann-Whitney U test was applied for skewed data.

To control for confounding variables, Propensity Score Matching (PSM) was used to balance the baseline characteristics between the ondansetron and non-ondansetron groups. The propensity score was estimated using logistic regression, where the treatment assignment (ondansetron or not) was modeled as a function of observed covariates, including age, gender, comorbidities, and stroke severity. Patients in the treatment group (ondansetron) were matched with those in the control group (non-ondansetron) based on the propensity score using nearest-neighbor matching with a caliper width of 0.05. After matching, the covariates between the two groups were assessed for balance using the standardized mean difference (SMD). An SMD of less than 0.1 indicated adequate balance.

Multivariable Cox regression and Kaplan-Meier survival curves were utilized to analyze the relationships between ondansetron usage and 30-day and 60-day mortality rates. Analyses were performed on specific subgroups to assess the consistency of ondansetron treatment’s effect on 30-day and 60-day mortality across various subgroups, including gender, race, myocardial infarction, chronic pulmonary disease, hypertension, norepinephrine administration, and mechanical ventilation. Fewer than 15% of data were absent for study variables; additional information is provided in Supplementary Table S2. Missing data were imputed utilizing the “mice” package in R software. The “mice” method was selected due to its efficacy in handling both continuous and categorical variables. In contrast to single imputation methods, it produces multiple imputed datasets, yielding more dependable estimates and better addressing uncertainty.

All statistical evaluations were performed using R software (version 4.4.1). A p-value of less than 0.05 was regarded as statistically significant.

## Results

### Basic characteristics

Before Propensity Score Matching (PSM), we analyzed 5,297 stroke patients, among whom 3,296 were in the ondansetron treatment group and 1,371 were in the non-ondansetron treatment group. Compared with the non-ondansetron group, patients in the ondansetron group were younger (median age: 70.68 vs. 74.53; P < 0.001) and had a higher proportion of Caucasians (72.50% vs. 68.49%; P = 0.005). Additionally, the ondansetron treatment group had significantly higher comorbidity rates, including chronic pulmonary disease and sepsis (all P values <0.05). Meanwhile, we found significant differences in intervention measures between the two groups (all P values <0.05) in the ondansetron group, including the use of vasoactive drugs (epinephrine, norepinephrine), mechanical ventilation (MV), and continuous renal replacement therapy (CRRT).

**FIGURE 2:**
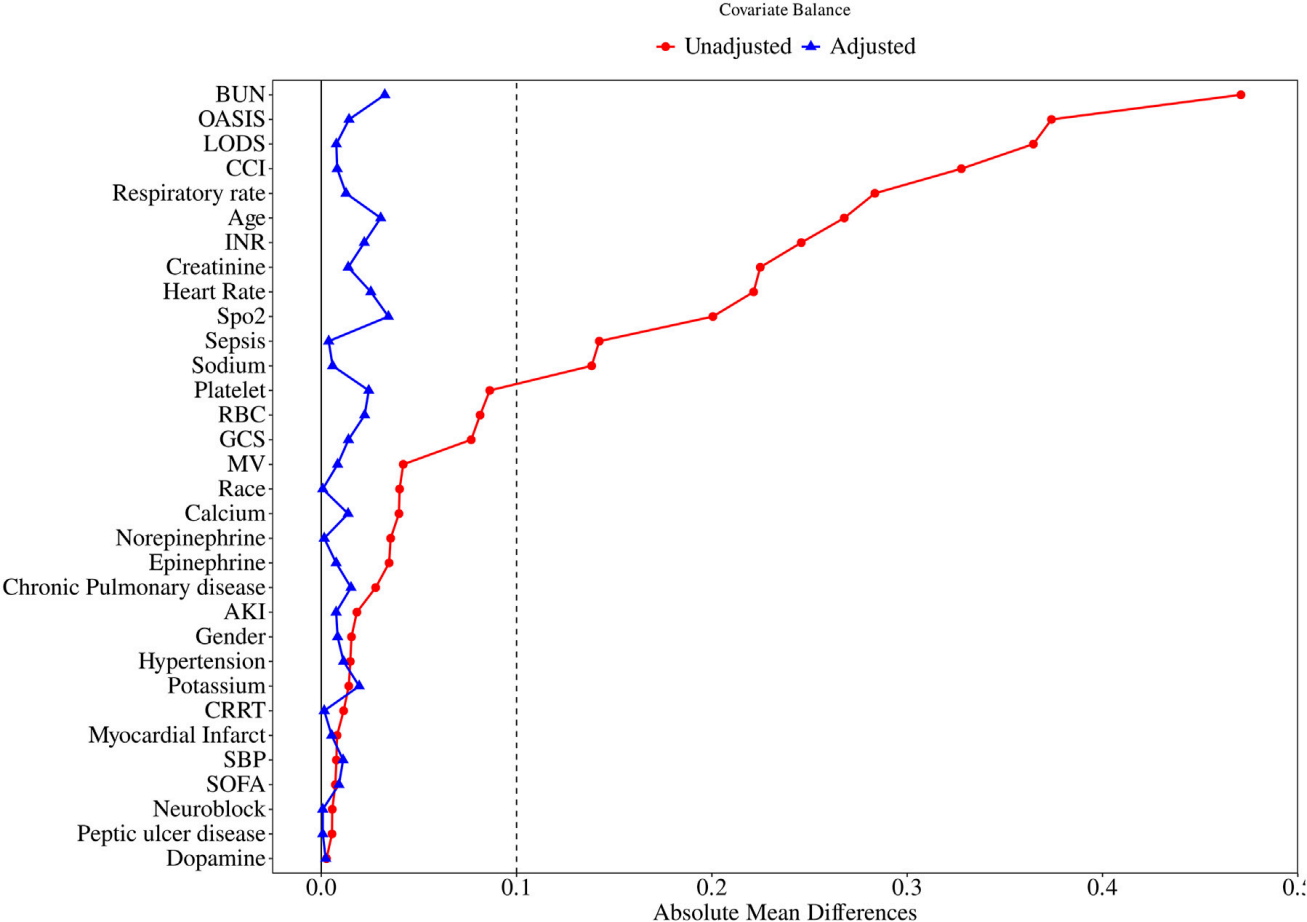
Standardized mean difference of variables before and after propensity score matching. SMD, standardized mean difference; BUN, blood urea nitrogen; OASIS, Original Acute Stroke Imaging Study; LODS, Logistic Organ Dysfunction Score. CCI, Charlson Comorbidity Index; INR, International Normalized Ratio; SpO2, blood oxygen saturation; RBC, Red Blood Cell; GCS, Glasgow Coma Scale; MV, mechanical ventilation; AKI, Acute Kidney Injury; CRRT, Continuous Renal Replacement Therapy; SBP, Systolic Blood Pressure; SOFA, Sequential Organ Failure Assessment.

After PSM, 1,314 pairs of matched patients were obtained, and the baseline characteristics between the two groups were well balanced (Table 1; Figure 2).

**TABLE 1:** Basic characteristics.

Variable	Before PSM					After PSM				
	Total (n = 5297)	No (n = 3926)	Yes (n = 1371)	*P*	SMD	Total (n = 2628)	No (n = 1314)	Yes (n = 1314)	*P*	SMD
Age (age)	73.52 (63.73, 82.54)	74.53 (64.71, 83.47)	70.68 (61.03, 79.51)	**<0.001**	−0.268	70.85 (61.01, 79.74)	70.61 (60.32, 79.91)	71.02 (61.65, 79.73)	0.439	0.031
Gender, n (%)				0.322					0.667	
Female	2412 (45.54)	1772 (45.13)	640 (46.68)		0.031	1203 (45.78)	596 (45.36)	607 (46.19)		0.017
Male	2885 (54.46)	2154 (54.87)	731 (53.32)		−0.031	1425 (54.22)	718 (54.64)	707 (53.81)		−0.017
Race, n (%)				**0.005**					0.965	
white	3683 (69.53)	2689 (68.49)	994 (72.50)		0.090	1909 (72.64)	954 (72.60)	955 (72.68)		0.002
other	1614 (30.47)	1237 (31.51)	377 (27.50)		−0.090	719 (27.36)	360 (27.40)	359 (27.32)		−0.002
Heart Rate (bmp)	82.00 (72.00, 95.00)	83.00 (72.00, 97.00)	80.00 (71.00, 91.00)	**<0.001**	−0.221	80.00 (71.00, 92.00)	81.00 (71.00, 92.00)	80.00 (71.00, 91.00)	0.672	−0.025
SBP (mmHg)	129.00 (111.00, 147.00)	129.00 (111.00, 148.00)	128.00 (112.00, 147.00)	0.809	−0.008	129.00 (112.00, 147.00)	129.00 (112.00, 147.00)	128.00 (112.00, 147.00)	0.953	0.011
Respiratory rate (bmp)	18.00 (15.00, 22.00)	18.00 (15.00, 22.00)	17.00 (15.00, 20.00)	**<0.001**	−0.283	17.00 (14.50, 20.00)	17.00 (14.00, 21.00)	17.00 (15.00, 20.00)	0.785	−0.013
Spo2 (%)	98.00 (96.00, 100.00)	98.00 (96.00, 100.00)	99.00 (96.00, 100.00)	**<0.001**	0.201	99.00 (96.00, 100.00)	98.00 (96.00, 100.00)	99.00 (96.00, 100.00)	0.194	0.034
RBC (10^9^/L)	3.67 (3.12, 4.21)	3.68 (3.13, 4.23)	3.63 (3.10, 4.16)	**0.025**	−0.081	3.66 (3.11, 4.20)	3.68 (3.09, 4.24)	3.65 (3.12, 4.17)	0.510	−0.022
Platelet (10^9^/L)	194.00 (147.00, 254.00)	196.00 (147.00, 257.00)	189.00 (146.00, 246.00)	**0.032**	−0.086	191.00 (146.00, 247.00)	191.00 (145.00, 247.00)	191.50 (148.00, 248.00)	0.564	0.024
BUN (mg/dL)	19.00 (14.00, 31.00)	21.00 (14.25, 34.00)	17.00 (12.00, 24.00)	**<0.001**	−0.471	17.00 (13.00, 25.00)	18.00 (13.00, 25.00)	17.00 (12.00, 25.00)	0.229	−0.032
Creatinine (mg/dL)	1.00 (0.80, 1.40)	1.00 (0.80, 1.60)	0.90 (0.70, 1.20)	**<0.001**	−0.225	0.90 (0.70, 1.20)	0.90 (0.70, 1.30)	0.90 (0.70, 1.20)	0.201	−0.014
Sodium (mmol/L)	139.00 (136.00, 141.00)	139.00 (136.00, 142.00)	139.00 (136.00, 141.00)	**<0.001**	−0.138	139.00 (136.00, 141.00)	139.00 (136.00, 141.00)	139.00 (136.00, 141.00)	0.510	0.006
Potassium (mmol/L)	4.10 (3.70, 4.50)	4.10 (3.70, 4.50)	4.10 (3.80, 4.50)	0.452	−0.014	4.10 (3.70, 4.50)	4.00 (3.70, 4.40)	4.10 (3.80, 4.50)	0.068	0.019
INR	1.20 (1.10, 1.50)	1.20 (1.10, 1.50)	1.20 (1.10, 1.40)	**<0.001**	−0.246	1.20 (1.10, 1.40)	1.20 (1.10, 1.40)	1.20 (1.10, 1.40)	0.076	−0.022
SOFA	1.00 (0.00, 3.00)	1.00 (0.00, 3.00)	1.00 (0.00, 2.00)	0.375	0.007	1.00 (0.00, 3.00)	1.00 (0.00, 3.00)	1.00 (0.00, 2.00)	0.543	−0.009
GCS	15.00 (14.00, 15.00)	15.00 (14.00, 15.00)	15.00 (15.00, 15.00)	**0.014**	0.077	15.00 (15.00, 15.00)	15.00 (15.00, 15.00)	15.00 (15.00, 15.00)	0.308	−0.014
OASIS	32.00 (26.00, 38.00)	33.00 (27.00, 39.00)	30.00 (24.00, 36.00)	**<0.001**	−0.374	30.00 (25.00, 35.00)	30.00 (25.00, 35.00)	30.00 (24.00, 36.00)	0.791	0.014
LODS	4.00 (2.00, 6.00)	4.00 (2.00, 6.00)	3.00 (2.00, 5.00)	**<0.001**	−0.365	3.00 (2.00, 5.00)	3.00 (2.00, 5.00)	3.00 (2.00, 5.00)	0.600	−0.008
CCI	6.00 (4.00, 8.00)	6.00 (4.00, 8.00)	5.00 (4.00, 7.00)	**<0.001**	−0.328	5.00 (4.00, 7.00)	5.00 (4.00, 7.00)	5.00 (4.00, 7.00)	0.864	−0.008
Calcium (mg/dL)	8.50 (8.00, 9.00)	8.50 (8.00, 9.00)	8.50 (8.00, 8.95)	0.109	−0.040	8.50 (8.00, 9.00)	8.50 (8.00, 9.00)	8.50 (8.00, 9.00)	0.434	0.014
Myocardial Infarct, n (%)	1156 (21.82)	865 (22.03)	291 (21.23)	0.533	−0.020	573 (21.8)	290 (22.07)	283 (21.54)	0.741	−0.013
Chronic Pulmonary disease, n (%)	1338 (25.26)	1020 (25.98)	318 (23.19)	**0.041**	−0.066	604 (22.98)	292 (22.22)	312 (23.74)	0.354	0.036
Peptic ulcer disease, n (%)	122 (2.3)	96 (2.45)	26 (1.90)	0.244	−0.040	51 (1.94)	25 (1.90)	26 (1.98)	0.888	0.005
AKI, n (%)	3997 (75.46)	2981 (75.93)	1016 (74.11)	0.177	−0.042	1962 (74.66)	986 (75.04)	976 (74.28)	0.654	−0.017
Sepsis, n (%)	2537 (47.9)	2025 (51.58)	512 (37.35)	**<0.001**	−0.294	1015 (38.62)	510 (38.81)	505 (38.43)	0.841	−0.008
Hypertension, n (%)	4204 (79.37)	3131 (79.75)	1073 (78.26)	0.242	−0.036	2077 (79.03)	1046 (79.60)	1031 (78.46)	0.472	−0.028
Dopamine, n (%)	111 (2.1)	85 (2.17)	26 (1.90)	0.550	−0.020	55 (2.09)	29 (2.21)	26 (1.98)	0.683	−0.016
Epinephrine, n (%)	250 (4.72)	150 (3.82)	100 (7.29)	**<0.001**	0.134	172 (6.54)	91 (6.93)	81 (6.16)	0.430	−0.032
Norepinephrine, n (%)	889 (16.78)	695 (17.70)	194 (14.15)	**0.002**	−0.102	374 (14.23)	188 (14.31)	186 (14.16)	0.911	−0.004
Neuroblock used, n (%)	80 (1.51)	65 (1.66)	15 (1.09)	0.142	−0.054	31 (1.18)	16 (1.22)	15 (1.14)	0.857	−0.007
MV, n (%)	4147 (78.29)	3031 (77.20)	1116 (81.40)	**0.001**	0.108	2141 (81.47)	1076 (81.89)	1065 (81.05)	0.581	−0.021
CRRT, n (%)	157 (2.96)	128 (3.26)	29 (2.12)	**0.031**	−0.080	60 (2.28)	31 (2.36)	29 (2.21)	0.794	−0.010

A p-value < 0.05 indicates that the result is statistically significant.

### Primary outcome

**FIGURE 3:**
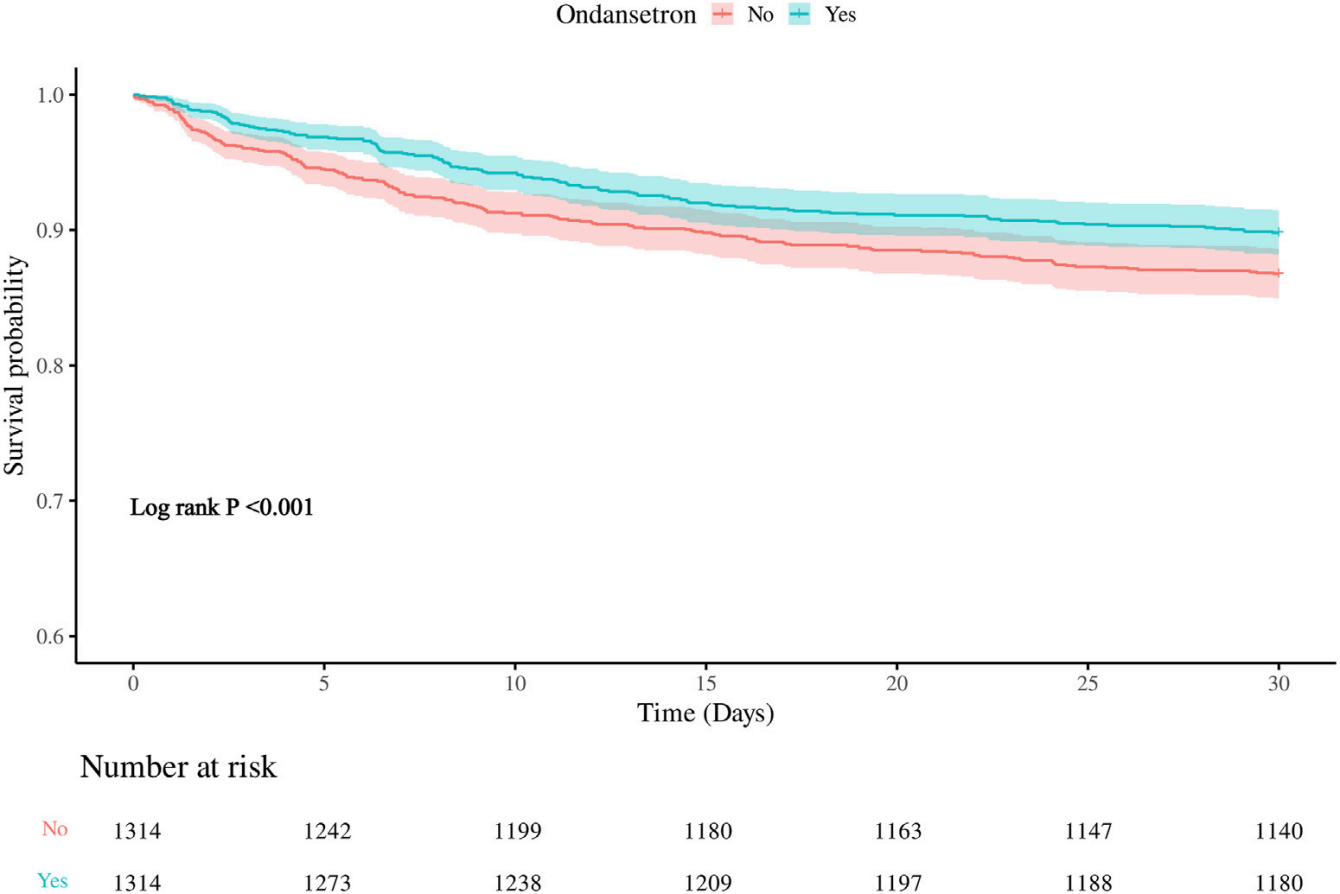
Kaplan–Meier survival curve for 30-daymortality. Ondansetron use was associated with improved 30-days urvival in the matched cohort.

The 30-day in-hospital mortality rate in the group treated with ondansetron was 27% lower than that in the group without ondansetron treatment. We established three models for analysis, with the 30-day mortality rate of patients as the evaluation criterion. The variables included in the models were selected based on their clinical relevance and the results from Supplementary Table S3, where covariates with p < 0.05 were included. We found that as the model changed from Model 1 to Model 3, the hazard ratio (HR) of patients who received ondansetron gradually decreased, and the P values also gradually became smaller (all <0.05). Moreover, the 95% confidence intervals consistently supported that the use of ondansetron was associated with a reduced 30-day in-hospital mortality rate. This indicates that in these three models, the use of ondansetron may be a factor contributing to the reduction of the 30-day in-hospital mortality rate, and this association became increasingly significant across different models (Table 2). The Kaplan-Meier survival curve further demonstrated that the survival curve of the ondansetron treatment group was improved (Figure 3).

**TABLE 2:** Association between Ondansetron use and mortality in stroke patients after PSM(30d).

Variables	Model1		Model2		Model3	
	HR (95%CI)	*P*	HR (95%CI)	*P*	HR (95%CI)	*P*
30-day hospital mortality						
Ondansetron						
No	1.00 (Reference)		1.00 (Reference)		1.00 (Reference)	
Yes	0.75 (0.60–0.94)	**0.013**	0.74 (0.59–0.93)	**0.010**	0.73 (0.59–0.92)	**0.007**

HR, Hazard Ratio, CI: Confidence Interval.

Model1, Crude.

Model2, Age, Gender, Race.

Model3, Adjust Age, Gender, Race, AKI, Heart Rate, GCS, Platelet, BUN, Norepinephrine, MV.

A p-value < 0.05 indicates that the result is statistically significant.

### Secondary outcome

**FIGURE 4:**
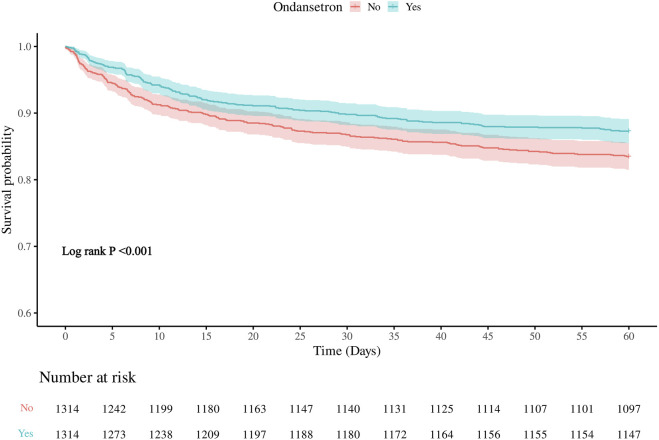
Kaplan–Meier survival curve for 60-daymortality. Ondansetron use was associated with improved 60-days urvival in the matched cohort.

Meanwhile, we also analyzed the 60-day in-hospital mortality rate of the patients. It was found that the 60-day mortality rate in the group treated with ondansetron was 28% lower than that in the group without ondansetron treatment. Similarly, as the model changed from Model 1 to Model 3, the hazard ratio (HR) of patients who received ondansetron gradually decreased, indicating that the use of ondansetron may be a factor contributing to the reduction of the 60-day in-hospital mortality rate (Table 3). The Kaplan-Meier survival curve further demonstrated that the survival curve of the ondansetron treatment group was improved (Figure 4).

**TABLE 3:** Association between Ondansetron use and mortality in stroke patients after PSM(60d).

Variables	Model1		Model2		Model3	
	HR (95%CI)	*P*	HR (95%CI)	*P*	HR (95%CI)	*P*
60-day hospital mortality						
Ondansetron						
No	1.00 (Reference)		1.00 (Reference)		1.00 (Reference)	
Yes	0.75 (0.61–0.92)	**0.005**	0.74 (0.61–0.91)	**0.004**	0.73 (0.60–0.90)	**0.003**

HR, Hazard Ratio, CI, Confidence Interval.

Model1, Crude.

Model2, Adjust, Age, Gender, Race.

Model3, Adjust Age, Gender, Race, AKI, Heart Rate, GCS, Platelet, BUN, Norepinephrine, MV.

A p-value < 0.05 indicates that the result is statistically significant.

### Subgroup analysis

**FIGURE 5:**
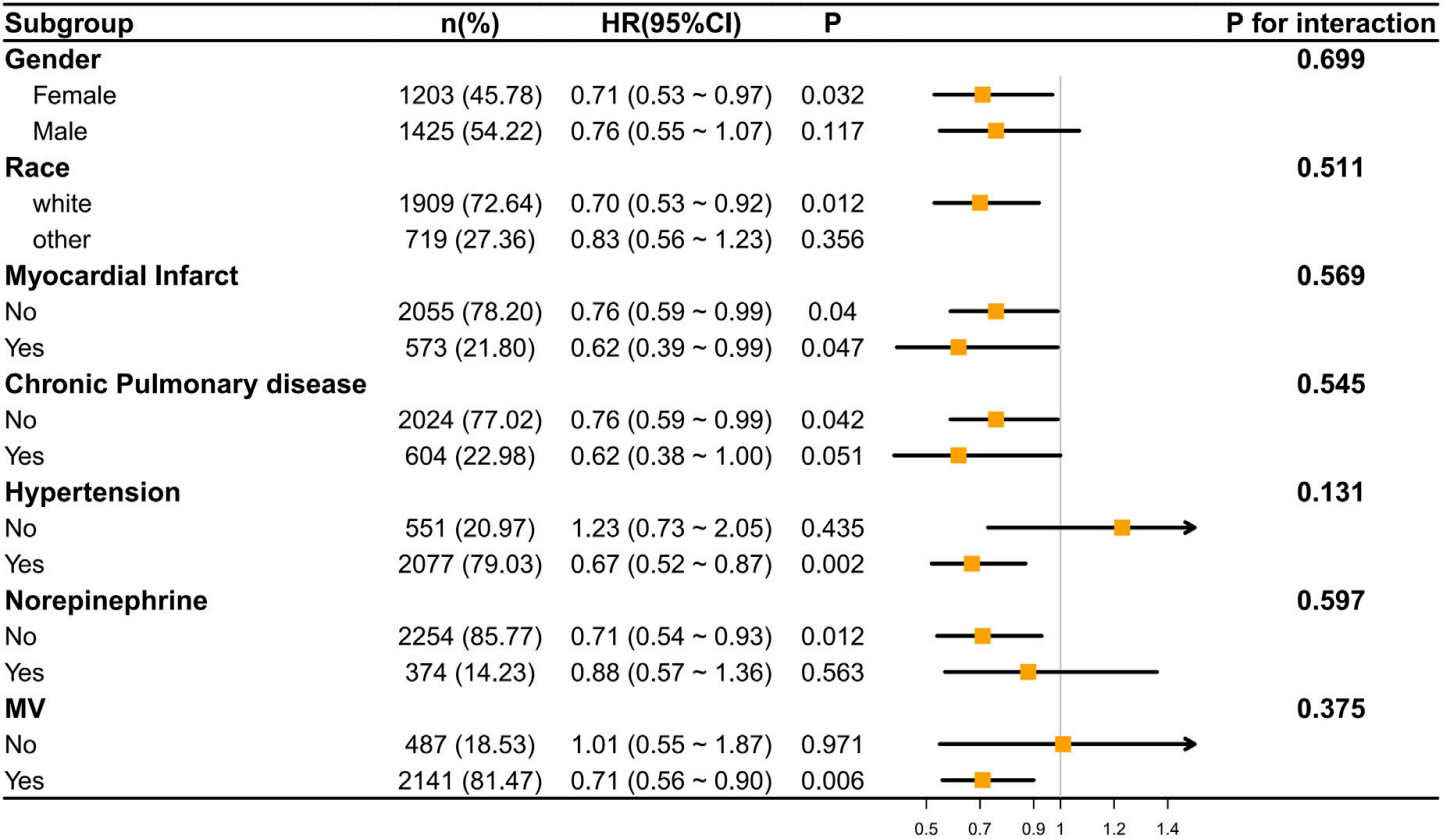
The association between ondansetron use and 30-day mortality in various subgroups.

**FIGURE 6:**
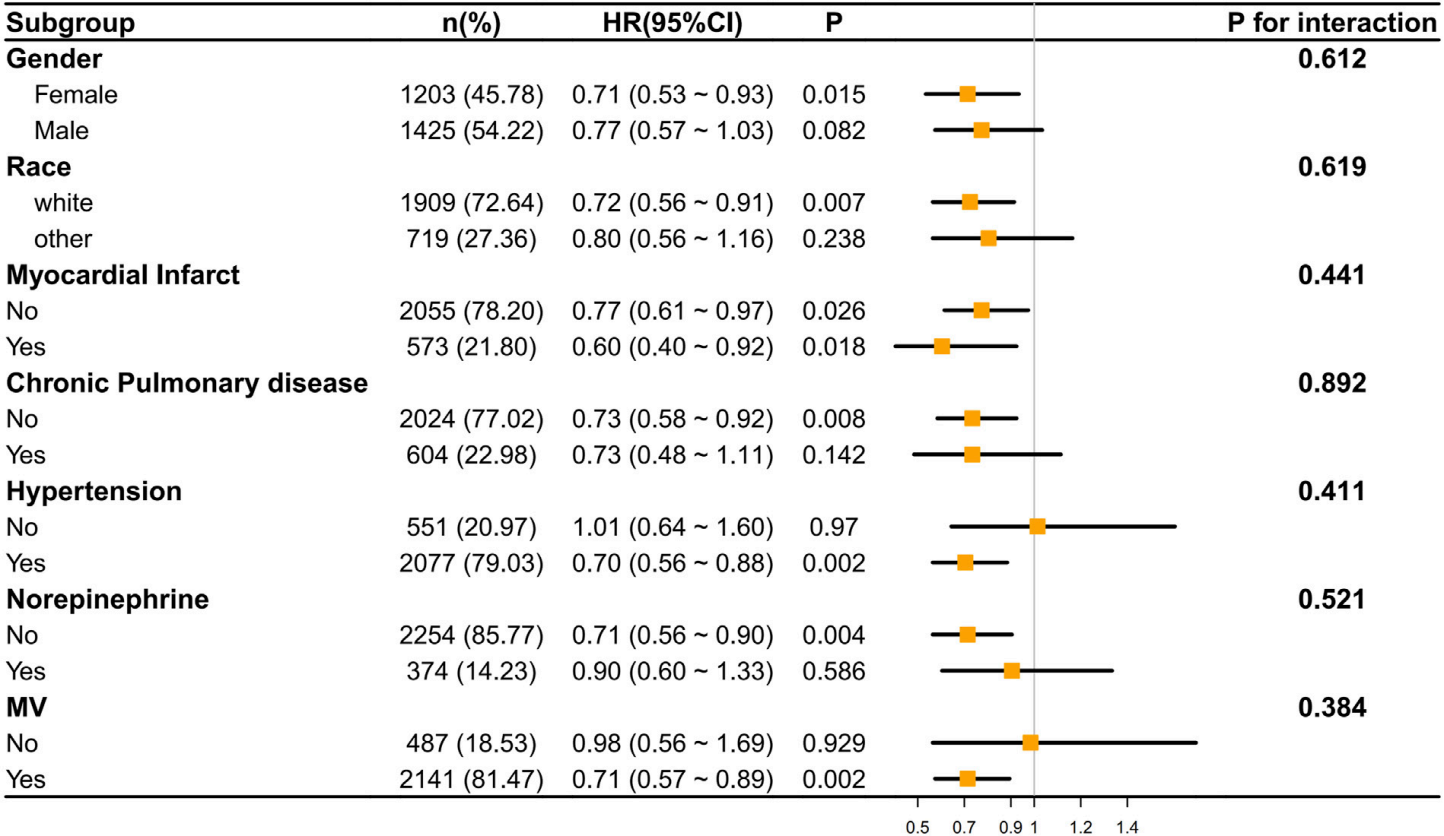
The association between ondansetron use and 60-day mortality in various subgroups.

We conducted an analysis of the correlation between ondansetron treatment in stroke patients and the decrease in 30-day/60-day mortality rates through subgroup analysis. It was found that in most subgroups, ondansetron treatment in stroke patients was associated with a decrease in 30-day/60-day mortality rates (p < 0.05), such as in the subgroups of gender (female), race (white), myocardial infarct, hypertension, norepinephrine use, and mechanical ventilation (MV). Meanwhile, we also found that no significant correlation was observed in the subgroups of gender (male), race (other), chronic pulmonary disease, and norepinephrine use (Figures 5, 6).

However, the P values of interaction in all subgroups were greater than 0.05, indicating that there was no statistically significant difference in the influence of different subgroups on the results. This implies that across different subgroups in terms of gender, race, disease status, etc., the overall trend of the effect was similar, and there were no obvious subgroup differences affecting the research outcomes.

## Discussion

In this extensive retrospective cohort study, a total of 6,645 stroke patients admitted for the first time to the ICU were initially identified. Following the exclusion of 0 patients under 18 years of age and 1,348 patients with an ICU stay shorter than 24 h, 5,297 patients qualified for inclusion in the study. These individuals were categorized into two groups: 3,926 patients received ondansetron while in the ICU, and 1,371 patients did not. By utilizing Propensity Score Matching (PSM), 2,628 patients were chosen for the final analysis (1,314 patients in each group). The findings indicated that ondansetron use was linked to a lower 30-day mortality rate among stroke patients admitted to the ICU (hazard ratio [HR] 0.73, 95% confidence interval [CI]: 0.59–0.92, P = 0.007). The secondary outcome suggested that ondansetron use was associated with a reduced 60-day mortality rate (HR 0.73, 95% CI: 0.60–0.90, P = 0.003). The subgroup analysis (with all P values of interaction exceeding 0.05) further supported these results.

Ondansetron, a 5-hydroxytryptamine type 3 (5-HT3) receptor antagonist, has long been used for managing nausea and vomiting, particularly in postoperative, chemotherapy, and critically ill patients. Recent studies have explored its potential beyond symptom management, with promising results in various critical care settings. Tao et al. found that ondansetron use in mechanically ventilated ICU patients was associated with a significant reduction in both in-hospital and 60-day mortality (Tao et al., 2023). Similarly, Yang et al. reported that ondansetron exposure in sepsis patients was linked to lower in-hospital and long-term mortality (Yang et al., 2023). Wang et al. also found a lower 30-day mortality rate in moderate-to-severe traumatic brain injury (TBI) patients treated with ondansetron (Wang et al., 2024). These findings suggest that ondansetron may offer protective benefits, likely through its modulation of inflammation and neuroprotection, though these studies remain observational and do not establish causality. While promising, concerns about ondansetron’s safety remain. Babi et al. raised potential neurological risks associated with high-dose ondansetron, citing a rare case of posterior reversible encephalopathy syndrome (PRES) in a patient who ingested excessive doses (Babi et al., 2016). Although rare, this adverse effect underscores the need for cautious administration of ondansetron, particularly in vulnerable populations. Despite the absence of direct evidence demonstrating that ondansetron lowers the mortality rate in stroke cases, the real-world data presented in our study add to the growing body of evidence suggesting a potentially beneficial role for ondansetron in improving stroke prognosis. Our analysis highlights that ondansetron use is significantly associated with a reduction in both 30-day and 60-day mortality rates in stroke patients, particularly in those who require intensive care.

In this study, we explored both the pathophysiology of stroke and the pharmacological mechanisms of ondansetron to investigate potential explanations for the observed reduction in mortality among stroke patients treated with ondansetron. First, stroke patients frequently experience vomiting, resulting from lesions affecting the brainstem’s vomiting center (Rosemergy et al., 2007) or reflexive vomiting triggered by nerve stimulation due to increased intracranial pressure (Hannoush et al., 2000). Aspiration of vomitus into the trachea and lungs can lead to aspiration pneumonia, a common complication, particularly among patients with altered consciousness. Aspiration pneumonia can cause pulmonary infections, exacerbate the patient’s condition, elevate systemic inflammatory responses, and, in severe cases, result in respiratory failure, thereby increasing mortality risk (Labeit et al., 2024). By mitigating vomiting, ondansetron can lower the risk of aspiration and subsequent pneumonia, indirectly decreasing mortality associated with pulmonary infection. Second, persistent vomiting can lead to significant loss of gastric juice, including crucial electrolytes like hydrogen and chloride ions (Moscona-Nissan et al., 2024). Electrolyte imbalances, such as hypochloremia and hypokalemia, can disrupt the function of vital systems, including the cardiovascular and neuromuscular systems. For example, hypokalemia can cause arrhythmias (Kim et al., 2023), potentially life-threatening (Tazmini et al., 2020). By alleviating vomiting, ondansetron aids in maintaining electrolyte and fluid balance, ensuring proper function of vital organs, and diminishing mortality related to electrolyte imbalances. Finally, recent studies have suggested a link between the 5-HT3 receptor and nerve injury following cerebral ischemia (Shayan et al., 2023). During ischemic stroke, the reduced blood supply triggers a cascade of complex pathophysiological responses in brain tissue. The 5-HT3 receptor may participate in these detrimental cascade reactions. Ondansetron’s antagonistic action on the 5-HT3 receptor might partially prevent or lessen these harmful responses, potentially providing a neuroprotective effect. While this mechanism requires further investigation, emerging evidence suggests that ondansetron contributes to neuroprotection post-stroke by modulating the neurotransmitter system, decreasing glutamate excitotoxicity, and preventing neuronal death associated with calcium overload, thereby indirectly lowering mortality (Fakhfouri et al., 2015; Kagami et al., 1992).

There are several intriguing subgroups identified in the subgroup analysis: no significant correlation was noted in the male gender subgroup, racial category (other), chronic pulmonary disease, and norepinephrine administration (all P > 0.05). Now, let’s examine the potential reasons for these findings. Firstly, pertaining to “gender”, a notable correlation was found in females but absent in males. The levels of hormones such as estrogen in females tend to be relatively high, and estrogen has the capability to regulate the neurotransmitter system, including the 5-hydroxytryptamine (5-HT) system, while ondansetron primarily exerts its action through selective blockage of the 5-HT3 recepto, estrogen may elevate the expression of 5-HT3 receptors in both the gastrointestinal tract and the central nervous system, allowing ondansetron to bind more effectively to these receptors in females, thereby enhancing its impact (Yan et al., 2014). Additionally, there are gender differences in liver drug - metabolizing enzymes, such as the cytochrome P450 system. Females may have lower activity of some liver enzymes. As a result, they metabolize ondansetron more slowly. This prolongs drug presence in the body, enhances receptor interaction, and boosts the therapeutic effect (Bartkowiak-Wieczorek et al., 2015). Secondly, concerning “race”, a significant correlation was observed in the white population but not in other racial categories, which could be linked to genetic polymorphism (Lu et al., 2014). In white populations, certain CYP2D6 gene subtypes speed up ondansetron metabolism, boosting its effectiveness. Genetic CYP2D6 variations cause differences in metabolism rates and metabolite activities, slower metabolism in white populations helps maintain optimal drug levels in blood, improving efficacy. Meanwhile there are racial differences in drug - transporter genes. P - glycoprotein affects ondansetron distribution. Gene variations in P - glycoprotein across races change its function. In white patients, P - glycoprotein’s transport mechanism may increase ondansetron accumulation near the 5 - HT3 receptor, enhancing treatment outcomes (Dey, 2006). Moreover, the subgroup analysis also indicated that “chronic pulmonary disease” did not exhibit any noteworthy correlation with the results. Patients suffering from chronic lung illnesses often have compromised respiratory functions. For instance, individuals with chronic obstructive pulmonary disease (COPD) experience ongoing airflow limitations (Negewo et al., 2015; McNeill et al., 2021; McKenzie et al., 2021). This restriction of airflow can induce changes in intrathoracic pressure, subsequently affecting blood perfusion and peristaltic activities in the gastrointestinal tract. Reduced gastrointestinal blood flow may hinder the absorption of drugs within this system. Individuals with chronic lung diseases frequently face hypoxia and carbon dioxide retention. Under conditions of hypoxia, the metabolic functioning of gastrointestinal mucosal cells can be altered, potentially diminishing their capability to actively transport medications. Additionally, carbon dioxide retention may lead to acidosis, thereby impacting the gastrointestinal environment’s pH, which is closely related to drug absorption (Davidson et al., 2016). For ondansetron, being a weakly alkaline compound, changes in gastrointestinal pH can influence its dissociation, ultimately impacting its absorption efficiency (Wolf, 2000). The final subgroup without significant correlation to the results was “norepinephrine use”. Norepinephrine primarily acts on α-adrenergic receptors, which can induce vasoconstriction, consequently reducing gastrointestinal blood flow (Nygren et al., 2003). This effect can slow the absorption processes in the gastrointestinal mucosa and prevent the timely achievement of effective therapeutic concentrations at the drug’s action site. Concurrently, vasoconstriction induced by norepinephrine may result in decreased blood flow to the liver (Cousineau et al., 1983). The liver plays a crucial role in drug metabolism, and many compounds, including ondansetron, necessitate liver metabolism. Diminished liver blood flow can adversely affect the functionality of drug-metabolizing enzymes, such as the cytochrome P450 enzyme system (Guengerich, 2019). This reduction may yield slower metabolic rates for ondansetron, making it challenging to maintain optimal therapeutic blood drug concentrations, thus negatively influencing its effectiveness.

It is important to note that ondansetron was most likely administered to stroke patients experiencing vomiting or nausea, a symptom often associated with more severe stroke presentations. This introduces a potential confounding factor, as patients who vomit may inherently differ from those who do not, potentially exhibiting more severe neurological deficits, higher intracranial pressure, or involvement of the brainstem. These factors, which could have influenced the decision to administer ondansetron, may themselves impact patient outcomes and remain unmeasured in our analysis. While propensity score matching helped balance observed baseline characteristics between the ondansetron and non-ondansetron groups, unmeasured confounders—such as stroke severity, occurrence of vomiting, or variations in consciousness—cannot be entirely ruled out. These unaccounted-for variables could still affect the results, suggesting that the association between ondansetron and reduced mortality may be influenced by factors not fully captured in this study. Therefore, while we observe a strong association between ondansetron use and improved survival, it is crucial to consider this indication bias when interpreting the findings.

It has been found that ondansetron can reduce the mortality rate of stroke patients with vomiting, which holds great significance for clinical practice. On one hand, it enables the optimization of clinical treatment strategies. Previously, ondansetron was mainly used for antiemetic purposes. This discovery has expanded its clinical application scope. Doctors can now more actively consider incorporating ondansetron into the comprehensive treatment plans for stroke patients with vomiting, rather than merely relieving the vomiting symptoms. For instance, for those stroke patients with vomiting who are at a high risk of death, the use of ondansetron may become a routine adjuvant treatment. Meanwhile, the clinical treatment procedures in hospitals may be altered accordingly. During the emergency and subsequent treatment of stroke patients, medical staff are likely to pay more attention to the observation and management of vomiting symptoms and initiate ondansetron treatment at an appropriate and early time. On the other hand, it can improve the prognosis of stroke patients. The reduction in the mortality rate means that more stroke patients with vomiting have the opportunity to survive, which brings great comfort to their families and can, to some extent, alleviate the economic burden on families. With the control of vomiting symptoms and the decrease in the mortality rate among stroke patients, the surviving patients have the chance to receive further rehabilitation treatment and reintegrate into social life. Simultaneously, it also expands the research directions in scientific research. This discovery will prompt researchers to conduct in-depth studies on the specific mechanisms by which ondansetron reduces the mortality rate of stroke patients with vomiting. For example, research may find that ondansetron activates a specific neuroprotective pathway, which in turn inspires researchers to develop new neuroprotective drugs based on this pathway.

This study reinforces the current research focus indicating that “the 5-HT3 receptor may be associated with nerve damage following cerebral ischemia” by utilizing real-world clinical data, although some limitations persist. Firstly, the median age of our sample was 70.85, and elderly patients typically exhibit poorer recovery and more comorbidities. Thus, our findings may be more applicable to older stroke patients and may not generalize to younger cohorts. Future research should aim for a more balanced age distribution to better evaluate ondansetron’s effects across different patient groups. Secondly, the data used in this study come from specific institutions and regions, which may limit the generalizability of our findings to other settings with different medical resources, healthcare systems, or disease prevalence. Additionally, despite using propensity score matching to reduce confounding, unmeasured variables such as stroke severity, genetic predispositions, and environmental factors could still influence the outcomes. Moreover, missing data (<15%) were handled, but their potential impact on our findings cannot be entirely ruled out. Future studies with standardized data reporting and more comprehensive data collection will help address these limitations and provide more robust conclusions. Finally, while this study offers valuable insights, the retrospective nature of the design limits our ability to establish causality, and prospective studies would be essential to validate and refine these findings. Future well-designed prospective studies could provide more robust conclusions on the role of ondansetron in mortality outcomes among stroke patients and help overcome the limitations inherent in this observational analysis.

## Conclusion

This study used 30-day and 60-day mortality rates of stroke patients in the ICU as evaluation criteria. The results indicate that treatment with ondansetron, a serotonin 5-HT3 receptor antagonist, is associated with improved short-term prognosis in stroke patients, particularly in the elderly. However, it is important to note that these findings reflect observational associations rather than causality. Future research, particularly randomized controlled trials, is necessary to validate these results and further explore the relationship between 5-HT3 receptors and nerve damage following cerebral ischemia.

## Data Availability

The datasets presented in this study can be found in online repositories. The names of the repository/repositories and accession number(s) can be found in the article/Supplementary Material.
