# Supplementary material for: Ondansetron: a potential intervention for improving clinical outcomes in stroke patients
Source: Front Pharmacol. 2025 Sep 9;16:1604117. doi: 10.3389/fphar.2025.1604117 (PMC12454419; doi:10.3389/fphar.2025.1604117)
Supplement: Supplementary file 1 [file Supplementaryfile1.docx]

**Table S1 Definitions and calculation methods of disease severity scores (SOFA, GCS, and CCI)**

| **Variable** | **Definition** | **Calculation method** |
| --- | --- | --- |
| SOFA Score | A score to assess organ dysfunction in critically ill patients, including respiratory, coagulation, liver, cardiovascular, renal, and neurological systems. | Each organ system (respiratory, coagulation, liver, cardiovascular, renal, neurological) is scored from 0 to 4, and the total score is the sum of individual organ scores (maximum 24). |
| Glasgow Coma Scale (GCS) | A scale to assess the level of consciousness, evaluating eye opening, verbal response, and motor response. | The eye opening, verbal response, and motor response are scored on scales from 1 to 4, 1 to 5, and 1 to 6 respectively. The total GCS score is the sum of these three components (3-15). |
| Charlson Comorbidity Index (CCI) | A comorbidity index used to classify comorbidities based on their impact on mortality risk, with a weight assigned to each condition. | The sum of weighted comorbidities based on predefined weights for conditions like myocardial infarction, diabetes, and chronic pulmonary disease. |

**Table S2 Missing variables and their percentages**

| **Column** | **Missing Percentage** |
| --- | --- |
| INR | 12.3466113 |
| Calcium | 8.4576175 |
| Glucose | 1.9444969 |
| RBC | 1.9067397 |
| Platelet | 1.8501038 |
| BUN | 1.6424391 |
| Potassium | 1.6235605 |
| Sodium | 1.5669247 |
| Creatinine | 1.5669247 |
| Respiratory rate | 0.2643005 |
| Spo2 | 0.2076647 |
| SBP | 0.1887861 |
| GCS | 0.1887861 |
| Heart Rate | 0.1699075 |
| SOFA | 0.1699075 |

**Table S3 univariable cox regression for 30-day all-cause mortality**

| Variables | *P* | HR (95%CI) |
| --- | --- | --- |
|  |  |  |
| Gender |  |  |
| F |  | 1.00 (Reference) |
| M | **<.001** | 0.68 (0.55 ~ 0.85) |
| Race |  |  |
| 1 |  | 1.00 (Reference) |
| 2 | **0.014** | 1.35 (1.06 ~ 1.71) |
| Age | **<.001** | 1.03 (1.02 ~ 1.04) |
| Heart Rate | **0.002** | 1.01 (1.01 ~ 1.02) |
| SBP | **0.015** | 1.01 (1.01 ~ 1.01) |
| Respiratory rate | **<.001** | 1.05 (1.03 ~ 1.07) |
| GCS | 0.188 | 0.97 (0.93 ~ 1.01) |
| OASIS | **<.001** | 1.09 (1.08 ~ 1.11) |
| LODS | **<.001** | 1.20 (1.16 ~ 1.24) |
| CCI | **<.001** | 1.21 (1.17 ~ 1.26) |
| AKI |  |  |
| No |  | 1.00 (Reference) |
| Yes | **<.001** | 1.71 (1.27 ~ 2.30) |
| Sepsis |  |  |
| No |  | 1.00 (Reference) |
| Yes | **<.001** | 1.79 (1.43 ~ 2.24) |
| Platelet | **0.008** | 1.01 (1.01 ~ 1.01) |
| BUN | **<.001** | 1.02 (1.01 ~ 1.02) |
| Creatinine | 0.055 | 1.07 (1.00 ~ 1.15) |
| Glucose | **<.001** | 1.01 (1.01 ~ 1.01) |
| Dopamine |  |  |
| No |  | 1.00 (Reference) |
| Yes | **<.001** | 3.05 (1.84 ~ 5.04) |
| Norepinephrine |  |  |
| No |  | 1.00 (Reference) |
| Yes | **<.001** | 2.56 (1.99 ~ 3.28) |
| Neuroblock used |  |  |
| No |  | 1.00 (Reference) |
| Yes | **<.001** | 3.23 (1.72 ~ 6.06) |
| MV |  |  |
| No |  | 1.00 (Reference) |
| Yes | **0.021** | 1.47 (1.06 ~ 2.03) |
| CRRT |  |  |
| No |  | 1.00 (Reference) |
| Yes | **<.001** | 4.86 (3.28 ~ 7.22) |
